# Unveiling the Sorption Properties of Graphene Oxide-M13 Bacteriophage Aerogels for Advanced Sensing and Environmental Applications

**DOI:** 10.1021/acsami.4c16202

**Published:** 2024-12-11

**Authors:** Kate Stokes, Yiwei Sun, Paolo Passaretti, Henry White, Pola Goldberg Oppenheimer

**Affiliations:** † School of Chemical Engineering, Advanced Nanomaterials Structures and Applications Laboratories, College of Engineering and Physical Sciences, University of Birmingham, Edgbaston, Birmingham B15 2TT, U.K.; ‡ Paragraf Ltd, Cambridge PB28 3EB, U.K.; § Institute of Cancer and Genomic Sciences, College of Medical and Dental Sciences, University of Birmingham, Birmingham B15 2TT, U.K.; ∥ BAE-Systems—Air Sector, Buckingham House, FPC 267, Filton, Bristol BS34 7QW, U.K.; ⊥ Healthcare Technologies Institute, Institute of Translational Medicine, Mindelsohn Way, Birmingham B15 2TH, U.K.

**Keywords:** graphene oxide, M13 bacteriophage, sorption, water, ethanol, acetone

## Abstract

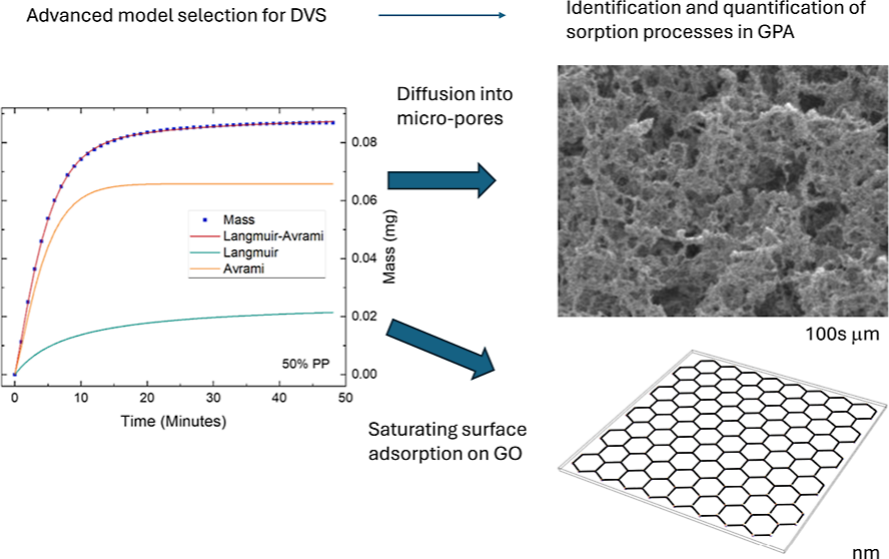

GraPhage13 aerogels (GPAs) are ultralow density, porous structures fabricated through the self-assembly of graphene oxide (GO) and M13 bacteriophage. Given GPA’s high surface area and extensive porous network, properties typically associated with highly adsorbent materials, it is essential to characterize its sorption capabilities, with a focus on unlocking its potential for advanced applications in areas such as biomedical sensing and environmental monitoring. Herein, the water, ethanol and acetone sorption properties of GPA were explored using dynamic vapor sorption (DVS). GPA was found to be highly hygroscopic, with a sorption capacity of 0.68 ± 0.02 g/g, double that of conventional desiccant silica gels and 20% higher than GO laminates. This remarkable sorption capacity, along with its sorption kinetics, was influenced by both GPA’s morphology and the strong interactions between the water molecules and the functional groups on the GO within GPA. The low hysteresis and stability of GPA during repeated sorption–desorption cycles highlight the reversibility of water sorption. While GPA shows lower capacity for ethanol and acetone, its tuneability presents opportunities for improving acetone sorption, and its ethanol sorption capacity exceeds that of similar carbon-based materials. These findings underscore GPA’s capability and versatility in vapor adsorption, paving the way toward its integration into graphene-based devices for sensing applications.

## 1 Introduction

The development of novel adsorbents with high sorption capacities and selective affinities for a range of vapors has been of an increasing interest due to the broad range of applications. For instance, highly hygroscopic materials capable of absorbing water from the environment have found applications in humidity control,^1^ moisture-enabled electric generation^2^ and freshwater generation,^3^ and adsorbents with an affinity for volatile organic compounds (VOCs) such as ethanol and acetone, have found applications in environmental monitoring^4,5^ and biomedical sensors.^6,7^ Activated carbon has been widely used as an adsorbent due to its high absorption capacity, resulting from its porosity, large surface area and hydrophilic functionalities, which can interact with the water and VOC molecules. However, it is known to exhibit certain limitations, including unwanted reactions which can limit its porosity and the mass loss which occurs during the regeneration of exhausted activated carbon.^8^ These drawbacks have highlighted the continued unmet need for developing novel carbon-based absorbents capable of retaining the advantages of activated carbon while simultaneously delivering cost-effectiveness and reusability.

**Figure 1:**
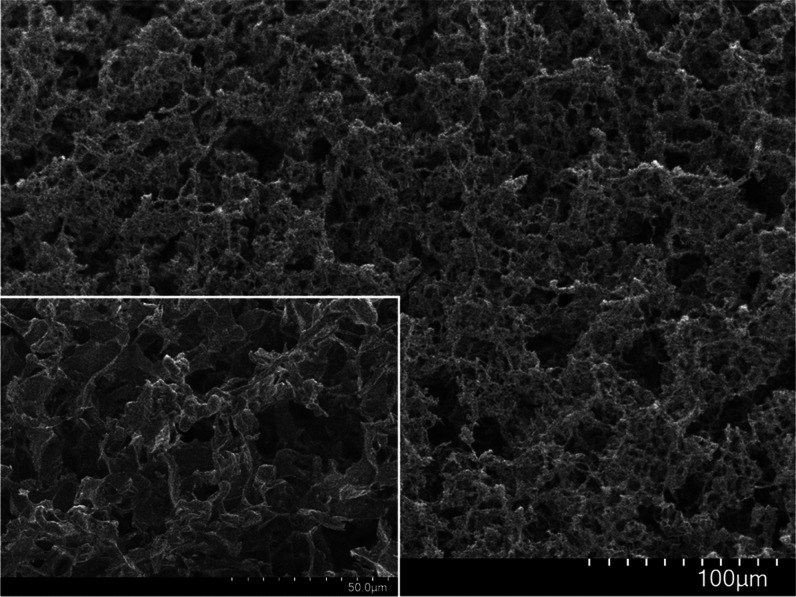
Scanning electron microscopy images of GraPhage13 aerogels showcasing the macro-porous structure. Inset: a zoomed-in view highlighting the ultralow density and high surface area of the structure.

Graphene oxide (GO) is an atomic layer of sp^2^ hybridized carbon atoms incorporating various oxygen-containing functional groups including carboxyl, carbonyl, epoxy and hydroxyl. Assembling GO into an aerogel combines the functional group interactions via GO with the porous, low density and high surface area of aerogel, enhancing the capabilities in adsorption and sensing.^9,10^ However, methods for producing aerogels with a GO precursor often rely on toxic and environmentally harmful reagents, or high-temperature and high-pressure conditions, which significantly increase production costs. In contrast, the self-assembly of GO with biomolecules presents a cost-effective, scalable and environmentally sustainable alternative.^11^ Existing graphene-based composites, such as graphene aerogels, reduced graphene oxide (rGO) aerogels, and graphene foams, have shown promise in adsorption applications due to their high surface areas and adsorption capacities. However, they face notable limitations that underscore the need for developing alternative materials like GPA. These challenges include cost-inefficient and toxic synthesis methods, limited scalability, and poor stability during cyclic use. For instance, graphene oxide foams demonstrate good adsorption of volatile organic compounds like acetone and ethanol but often exhibit hysteresis and lower water sorption capacities compared to GPA. Furthermore, traditional graphene-based composites are less environmentally friendly, relying on energy-intensive processes that limit their widespread adoption. In this regard, in a pioneering study, Passaretti et al. fabricated GO-based aerogels through the self-assembly of GO and M13 bacteriophage.^12^ M13 is a filamentous, nontoxic virus with a width of 6.6 nm and a length of 880 nm, consisting of circular-shaped single stranded DNA encapsulated by 2700 copies of the pVIII major coat protein, with minor coat proteins pIII and pVI at its head and pVII and pIX at its tail. The self-assembly process has been shown to generate the GraPhage13 aerogels (GPAs), which are macro-porous nanocomposites with an ultralow density (8.8 mg/cm^3^) and high surface area (325 m^2^/g)^12^ (Figure 1). Their self-assembly does not require high temperatures, pressures, or toxic reagents, and both GO and M13 can be generated on a large scale. The modular and self-assembling nature of GPA is inherently scalable. As demonstrated in our earlier work, the compatibility of GPA synthesis with standard chemical engineering techniques allows for its production at various scales without significant alterations to the process. This scalability makes GPA well-suited for adoption in commercial manufacturing. Furthermore, the low-cost precursors (GO and M13), combined with the absence of extreme conditions during synthesis, significantly reduce production costs. Our past studies also noted that the use of aqueous media and ambient conditions eliminates the need for expensive equipment, aligning GPA production with sustainable manufacturing practices. This enables the cost-effective, scalable, and environmentally friendly production of GPA, positioning it as a promising material for large-scale industrial applications.^13,14^ Furthermore, through the functionalization of GO, chemical/genetical modifications of M13, and incorporation of additional nanomaterials such as carbon nanotubes and gold nanoparticles into the porous structure, the characteristics of GPA could be effectively tailored for specific applications.^15,16^ These unique properties, combined with the low-cost, scalable and environmentally friendly production, renders the GPA an attractive, novel prospect for absorbents.

Herein, the sorption behavior of GPA for water, ethanol and acetone is comprehensively investigated through a series of in-depth dynamic vapor sorption (DVS) studies. GPA is found to exhibit a notably high water sorption capacity, surpassing that of conventional adsorbents. This performance is subsequently attributed to its extensive porous network, large surface area, multilayer adsorption and the strong interactions between water molecules and GO’s functional groups. While GPA’s sorption capacity for ethanol and acetone is lower than water, due to the finite nature of their multilayer adsorption and its weaker interactions with these solvents, its ethanol sorption capacity is highly competitive with similar carbon-based materials and the tunable properties of GPA offer a unique potential route for enhancing its acetone sorption capabilities. This exceptional sorption property of GPA further lay the platform for diverse applications including for instance, biomedical sensors and environmental monitoring. The study also paves the way for future research into exploring GPA’s affinity for different vapors for additional potential end uses such as environmental security^17^ and wastewater treatment.^18^ Moreover, the ability to fine-tune the properties of GPA provides avenues to optimize and enhance its sorption capabilities. This adaptability further underscores the versatility and potential of GPA in addressing a broad spectrum of environmental and technological challenges.

## 2 Results and Discussion

### 2.1 Water Sorption

**Figure 2:**
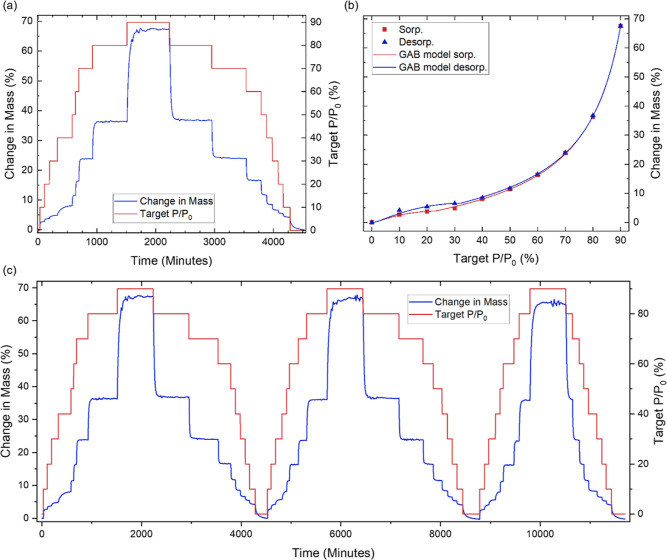
(a) Percentage change in mass of GPA over time as a function of the partial water vapor pressure variation between 0 and 90%. (b) Resulting isotherm illustrating the adsorption behavior. (c) Effect of subjecting GPA to three consecutive sorption–desorption cycles. The data facilitate the investigation of the kinetics, isotherms and hysteresis behaviors of water sorption on GPA.

The water sorption characteristics of GPA were investigated by monitoring the change in mass over time under varying partial pressures (PP), ranging from 0% to 90% in 10% increments (Figure 2a). At each PP step, the mass of GPA initially increased while it adsorbed water until either attaining the stop criterion, defined by a mass change below 0.002% per minute for a 10 min period, or reaching the maximum time limit of 12 h. For the PP steps between 10 and 80%, the stop criterion was achieved, indicating that the moisture content within the GPA had equilibrated with the surrounding environment.^19,20^ At 90% PP, GPA exhibited a mass change of 68 ± 2%. This is equivalent to a sorption capacity of 0.68 ± 0.02 g/g, more than double the capacity of conventional desiccant silica gels, and exceeds that of GO laminates by approximately 20%.^21^ Furthermore, in comparison to other porous carbon-based materials, GPA exhibits a comparable or superior water sorption capacity.^22,23^ The highly hygroscopic nature of GPA results from its extensive pore network and high specific surface area, creating a large volume for adsorption. The functional groups on GO within GPA provide specific binding sites for the water molecules through hydrogen bonding and electrostatic interactions, leading to stronger and more extensive interactions with the adsorbate, thereby enhancing its sorption capacity.^21,24^ Additionally, the stop criterion was not achieved at 90% PP and the maximum time limit was reached, suggesting that its sorption capacity of GPA may even exceed 0.68 ± 0.02 g/g, providing an avenue for future investigations. Throughout the experiments, no condensation or dripping of liquid water was observed. This confirms that the adsorption processes involve vapor-phase adsorption within the porous structure of GPA, rather than surface condensation leading to liquid formation.

The equilibrium mass recorded at the end of each PP step was subsequently used to generate the sorption–desorption isotherms (Figure 2b). Various models, including the Langmuir, Freundlich, Temkin, Brunauer–Emmett–Teller (BET), Guggenheim–Anderson–de Boer (GAB), and dual-site Langmuir–Freundlich (DLR) models, were applied to the isotherm data. The optimal fit was determined by the model with the lowest Bayesian information criterion (BIC) value.^25,26^ For water sorption by GPA, the Guggenheim–Anderson–de Boer (GAB) model was established as the most suitable for the isotherm representation.

The isotherm was classified as type II, characteristic of sorption by macroporous adsorbents with unrestricted monolayer–multilayer adsorption. Type II isotherms typically show an initial rapid increase in mass, reflecting the progressive formation of a monolayer of adsorbate on the adsorbent. This is followed by an inflection point, signaling the completion of monolayer sorption and the onset of multilayer adsorption. A more gradual curve suggests a smoother, overlapping transition between monolayer and multilayer adsorption. As adsorption continues, the mass increases as the multilayer thickness grows, with no apparent saturation point.^27^

The initial increase in mass between 0 and 10% suggests the formation of a monolayer of water on the surface of GPA, driven by adsorbent–adsorbate interactions. Specifically, the strongest interactions arise from the hydrogen bonding between the water molecules and the polar hydroxyl groups on GO.^28^ This is followed by a gradual increase in mass between 10% and 30% PP, indicating the gradual transition from monolayer to multilayer adsorption. The latter is facilitated by hydrogen bonds between water molecules in the environment and those adsorbed onto GPA. Beyond 40% PP, the mass of GPA increases more rapidly, suggesting that sorption becomes dominated by indefinite multilayer adsorption.^29,30^ To note, deriving an activation energy for the multilayer adsorption process is not feasible with the current data. Activation energy determination requires temperature-dependent kinetic measurements to relate the rate of adsorption to temperature. Since our study was conducted at a constant temperature and without kinetic measurements over a range of temperatures, the activation energy was not extracted from the data. Future studies involving temperature-dependent kinetic measurements could provide further insights into the energetics of the multilayer adsorption process.

Between 10 and 30% PP, during the transition between monolayer and multilayer sorption, a low level of hysteresis was observed. This can be attributed to the strong hydrogen bonds between the water molecules and hydroxyl groups on GPA, possessing a high binding energy. Breaking these bonds during desorption requires more energy than forming them during sorption, leading to the observed hysteresis.^31^ However, this hysteresis is minimal and only observed in the 10–30% PP range, reflecting the reversibility of the water sorption and suggesting that the overall sorption–desorption processes induce little structural change to GPA.^32^

To further investigate this, GPA was subjected to three consecutive sorption–desorption cycles (Figure 2c), where it exhibited excellent repeatability and stability. The most significant difference between the cycles is observed at 90% PP, where the mass decreases by 1.20% from cycle 1 to cycle 2 and by 1.51% from cycle 2 to cycle 3. This most probably originates from small structural changes, such as the stresses and strains exerted on the GPA during sorption–desorption, causing a fraction of the pores to collapse, or volumetric shrinkage due to the attractive bridging of the water molecules across the pores.

The third sorption–desorption cycle in Figure 2c appears shorter than the preceding cycles. This observation is attributed to the material’s behavior under repeated cycling rather than experimental inconsistency. Each stage’s stop criterion was defined as a mass change of less than 0.002% per minute, stable over 10 min. With each cycle, minor structural changes such as slight pore collapse or volumetric shrinkage may occur due to the stresses exerted during sorption–desorption.^33^ These changes can result in a quicker attainment of equilibrium in subsequent cycles, leading to shorter cycle durations. At the highest partial pressure (90% PP), the mass change during the third cycle exhibits increased noise and a subtle slope during the hold time. This is attributed to the gradual water sorption of GPA toward an equilibrium, which is not achieved before the maximum time limit and the increased sensitivity of the balance at high humidity levels. The fluctuations are minimal and stop before the transition to the subsequent partial pressure stage, thus not significantly affecting the overall adsorption capacity or data interpretation. While we observed excellent repeatability and stability over three cycles, we acknowledge that assessing mass loss over only three cycles provides a limited view of long-term cycling stability. However, our primary focus has been on investigating the kinetics and isotherm behaviors of GPA. In applications such as gas or molecular sensing in medical devices, where components are often disposable or intended for single use, extensive long-term cycling may not be critical. Future studies will explore the durability of GPA over a larger number of cycles to evaluate its potential for reusable applications.

To gain insights into the sorption kinetics, a range of kinetic models such as the Langmuir, pseudo-second-order, Avrami, Weibull and intraparticle diffusion models, were applied to each sorption and desorption stage (Figure 2a).^34^ The model with the lowest BIC value was considered the best fit, allowing for the analysis of the mechanisms driving water sorption kinetics in GPA. When the difference in BIC values was below 6, indicating that the models were statistically close, the preferable model was selected based on the physical significance of the fitted values and their consistency with the parameters of preceding or subsequent segments.^35,36^ Given the complexity of GPA sorption characteristics, arising from its porous structure and GPA-water interactions, no single model provided an optimal fit across many of the PP stages. Instead, hybrid models combining two different kinetic sorption models were required.^37^

**Figure 3:**
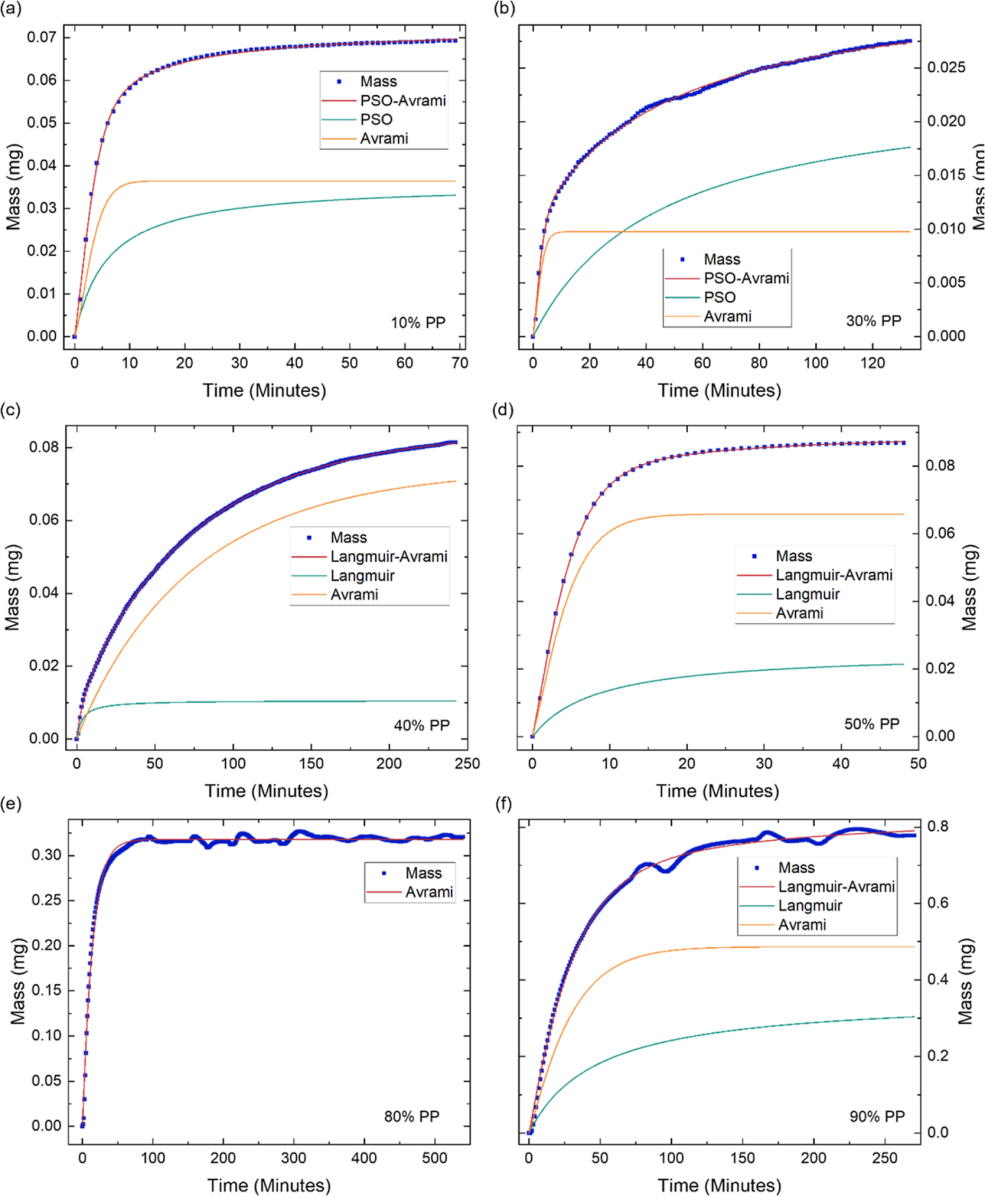
Water sorption kinetics of GPA demonstrating the increase in mass over time at different partial pressures (PP) of (a) 10% PP, (b) 30% PP, (c) 40% PP, (d) 50% PP, (e) 80% PP and (f) 90% PP. The starting mass and time are shifted to zero and the fit of the hybrid models and their individual components are displayed. The presented data enables the in-depth investigation of GPA’s water sorption kinetics.

When subjecting GPA to PPs between 10 and 30%, the kinetics were best described by a hybrid of the pseudo-second order (PSO) and the Avrami models (Figure 3a,b). The Avrami model exhibited a rapid initial sorption, while the PSO model showed a more gradual sorption process. The PSO model typically describes sorption dominated by interactions between the adsorbent and adsorbate. In this context, the PSO model represents the gradual formation of strong hydrogen bonds between the water molecules and the highly polar functionalities on GO, particularly its hydroxyl groups.^26,28^ As the PP increases from 10% to 30%, the contribution of the PSO component increases. The rising PP allows the water vapor to penetrate deeper into the porous structure, exposing more functional groups for the water molecules to bind with.^38,39^ The Avrami model is associated with the nucleation and growth of different phases. The Avrami equation, which describes the dependence of the nucleation and growth of new phases, can be employed to gain insights into the sorption mechanism. The Avrami exponent, *n*, is a representation of the dimensionality of the growth of the new phase and generally lies between 1 ≤ *n* ≤ 4, with 1 ≤ *n* ≤ 2 representing one-dimensional (1D) growth, 2 ≤ *n* ≤ 3 representing two-dimensional (2D) growth, and 3 ≤ *n* ≤ 4 representing three-dimensional (3D) growth.^40^ Throughout the water sorption process, the Avrami exponent consistently lies within the 1 ≤ *n* ≤ 2 range, with an average value of 1.1 ± 0.1. This suggests that water sorption is 1D and homogeneous across GPA, with the likelihood of adsorption occurring being uniform across all areas within a given time interval.^41^

At 40% PP, the optimal model shifts to the Langmuir-Avrami model (Figure 3c), which describes monolayer sorption of the adsorbate onto the surface and within the pores of the adsorbent, with sorption kinetics proportional to the concentration of adsorbed molecules. The shift from the PSO model to the Langmuir model, and its low contribution toward the sorption kinetics, indicates the saturation of GO’s hydroxyl groups, the completion of monolayer sorption and the transition to multilayer sorption, consistent with Figure 2b. The significant^42,43^ contribution of the Avrami model indicates the presence of homogeneous layer-by-layer water sorption, facilitated by hydrogen bonds between the water molecules already adsorbed on the surface of GPA and those in the surrounding environment.^41,44^ Between 50% and 70% PP, the Langmuir-Avrami model remains optimal (Figure 3d), however the contribution of the Langmuir component increases with PP, due to the increasing water–water interactions as sequential layers of water molecules form. At 80% PP, the Avrami model alone best represents the sorption kinetics (Figure 3e), reflecting a more consistent, organized growth of multilayers, where each layer adsorbs at a similar rate. However, at 90% PP, the optimal model transitions back to the Langmuir–Avrami hybrid model (Figure 3f). The renewed dependence of the sorption kinetics on the concentration of water previously adsorbed onto GPA suggests that the sorption is becoming constrained by the diffusion into the porous structure, reflecting the progressive occupation of water within the pores.^42^

**Figure 4:**
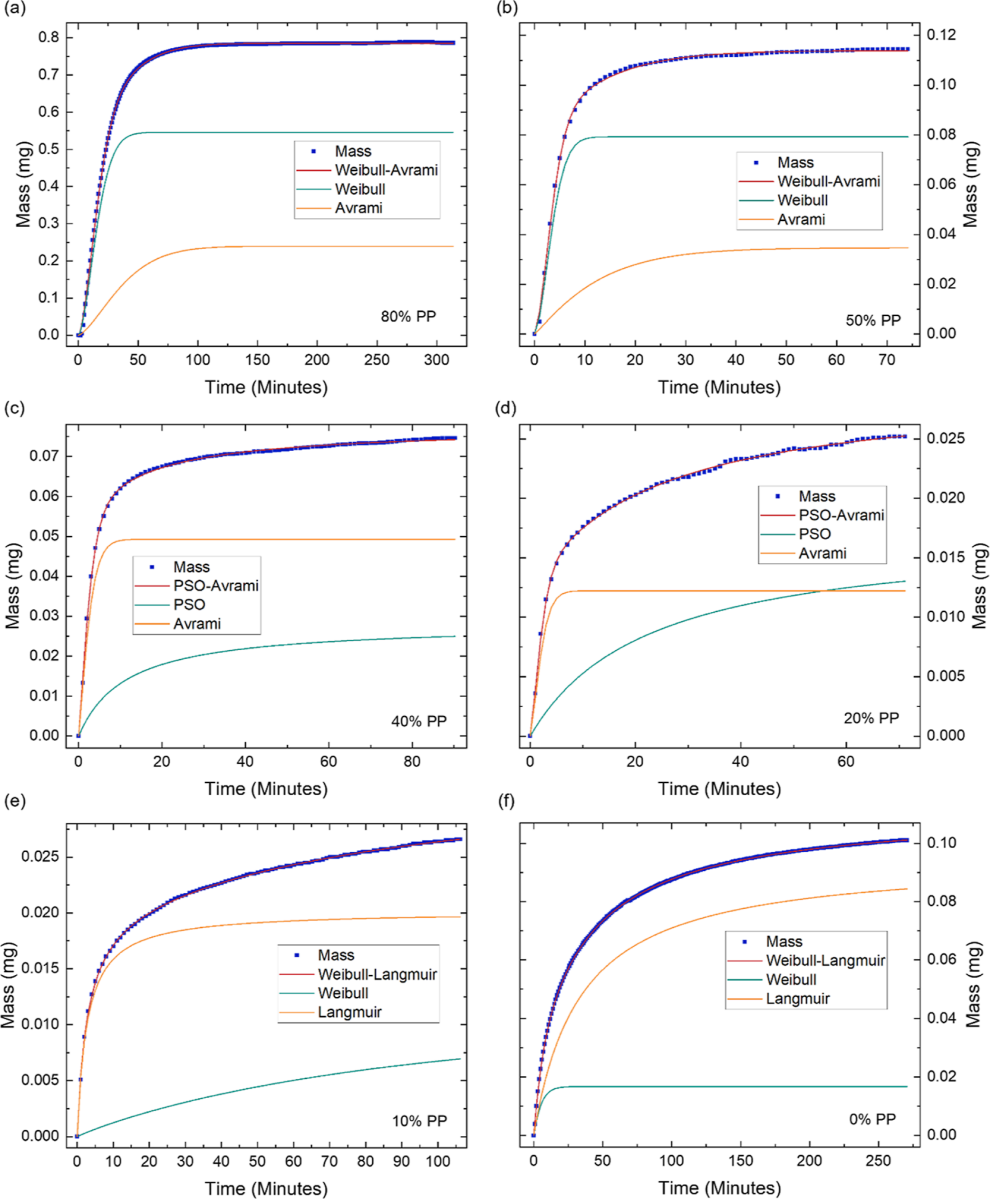
Water desorption kinetics of GPA, illustrating the mass decrease over time at different partial pressures of (a) 80% PP, (b) 50% PP, (c) 40% PP, (d) 20% PP, (e) 10% PP and (f) 0% PP. The initial mass and time are shifted to zero and the mass values are multiplied by (−1) for easier comparison with the adsorption kinetics. The fit of the hybrid models is displayed along with the individual components. The presented data allows for the in-depth investigation of GPA’s water desorption kinetics.

Following the in-depth examination of the adsorption kinetics, the desorption process was subsequently analyzed to interpret the underlying mechanisms governing the release of adsorbed water. The desorption stages between 80 and 50% PP are best described by the Weibull–Avrami hybrid model (Figure 4a,b). The Weibull component exhibits rapid desorption which rapidly plateaus, whereas the Avrami component demonstrates more gradual desorption. The Weibull model is often applied to describe the kinetics of water desorption, representing it as a series of probabilistic events and effectively captures desorption from heterogeneous surfaces.^45^ The heterogeneity arises from water being confined within pores of varying depths and volumes within the porous structure. As a result, water molecules exhibit different probabilities of desorption based on their varying capacities to diffuse out of these pores. On the other hand, the Avrami component represents the uniform release of water molecules, indicating the gradual desorption of water layers from the multilayer structure on GPA’s surface.

Between 40% and 30% PP, desorption is best described by the hybrid PSO-Avrami model (Figure 4c), where the Avrami component dominates, exhibiting a more rapid desorption. The transition from the Weibull model to the PSO model suggests that the water molecules with lower binding affinities have desorbed. The desorption of the water molecules with higher binding energies, such as those bound to GO’s hydroxyl groups, are better captured by the PSO model. However, the desorption is dominated by the layer-by-layer release of water molecules. At 20% PP, the PSO component gradually becomes more dominant than the Avrami component (Figure 4d), indicating that the desorption of the strongly bound water molecules becomes the primary desorption mechanism.

Lowering the PP further to 10%, the optimal model transitions to the Weibull–Langmuir model (Figure 4e). The Langmuir component is the faster and more dominant component, while the Weibull component shows a more gradual increase. The Langmuir model describes the desorption of the water molecules from the few remaining occupied energetically favorable states, with the probability of desorption governed by the occupancy of these sites. The Weibull model, on the other hand, accounts for the variability in binding site strengths, originating from some of the less energetically favorable sites still being occupied, and water remaining constrained within the porous structure. At 0%, the Weibull–Langmuir model remains optimal. However, the Weibull component rapidly plateaus (Figure 4f), indicating that the desorption is dominated by the release of water molecules from the high-energy binding sites. Despite the distinct kinetic pathways of adsorption and desorption, the close agreement between equilibrium points and the minimal hysteresis observed (Figure 2b), together with the demonstrated stability of GPA under consecutive sorption–desorption cycles (Figure 2c), suggest that these differences in kinetics do not adversely affect the reversibility of water sorption by GPA.

### 2.2 Ethanol Sorption

**Figure 5:**
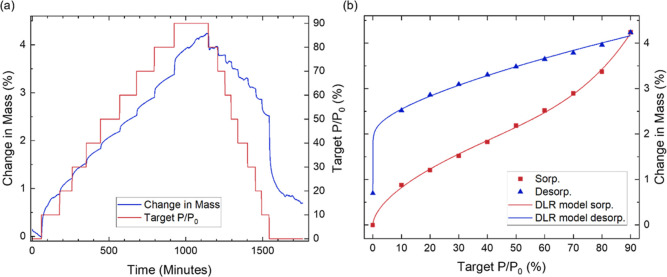
(a) Percentage change in mass of GPA over time as the partial pressure of ethanol vapor is varied between 0 and 90%. (b) Resulting isotherm illustrating the adsorption behavior. The presented data facilitate the investigation of the kinetics, isotherms and hysteresis behaviors of ethanol sorption on GPA.

Following the investigations into the water sorption in GPA, the ability of GPA to adsorb organic solvents, specifically ethanol and acetone, was further studied. GPA shows a reduced capacity for ethanol compared to water, with a sorption capacity of 4.2 ± 0.2% (0.042 ± 0.002 g/g) at 90% PP (Figure 5a). The bonding of the ethanol molecules to GO via their hydroxyl groups leaves the nonpolar alkyl groups on the surface of GPA. Bonding between the adsorbed and introduced ethanol molecules is predominately governed by weak van der Waals forces, reducing the probability of strong bonding or interactions between the ethanol molecules and reducing its sorption capacity.^28,46^

The lower sorption capacity for ethanol compared to water also leads to the stop criterion being attained more rapidly, hence the shorter hold times consistently observed across all samples. However, GPA exhibits a similar or improved ethanol sorption capacity compared to other porous carbon-based materials.^47,48^ This can be attributed to the combination of high surface area and the presence of oxygen-containing functional groups, which facilitate strong interactions with ethanol molecules. Unlike traditional activated carbons, which rely primarily on physical adsorption, GPA’s structure supports both physical and chemical interactions, leading to more efficient sorption.^49^

The ethanol sorption behavior of GPA exhibits a type IVa isotherm, typically observed in mesoporous adsorbents (Figure 5b). Type IV isotherms are indicative of sorption behavior driven by adsorbent–adsorbate interactions and capillary condensation, where the high PP initiates the condensation of vapor within the pores.^50^ For type IVa isotherms in particular, capillary condensation leads to hysteresis between the sorption and desorption curves and irreversible sorption of the adsorbate, shown by the 0.7% mass change between the beginning of the sorption cycle and the end of the desorption cycle. This occurs when the pores exceed a critical width. Modeling the pores as cylinders open at both ends, the meniscus during adsorption is cylindrical, while during desorption it becomes hemispherical. Since the radius of curvature for the hemispherical meniscus is half that of the cylindrical meniscus, the evaporation pressure is much lower than the condensation pressure, leading to the observed hysteresis. This likely occurs for GPA as its pores are macro-porous in nature rather than mesoporous.^12,27,51^ Furthermore, by minimizing the BIC, the dual-site Langmuir–Freundlich (DLR) model was identified as the best fit for the isotherm. This model captures the nonideal monolayer adsorption stemming from the complex interactions between GPA and ethanol, leading to the formation of nonpolar alkyl groups on GPA’s surface that inhibit multilayer adsorption.^52^

**Figure 6:**
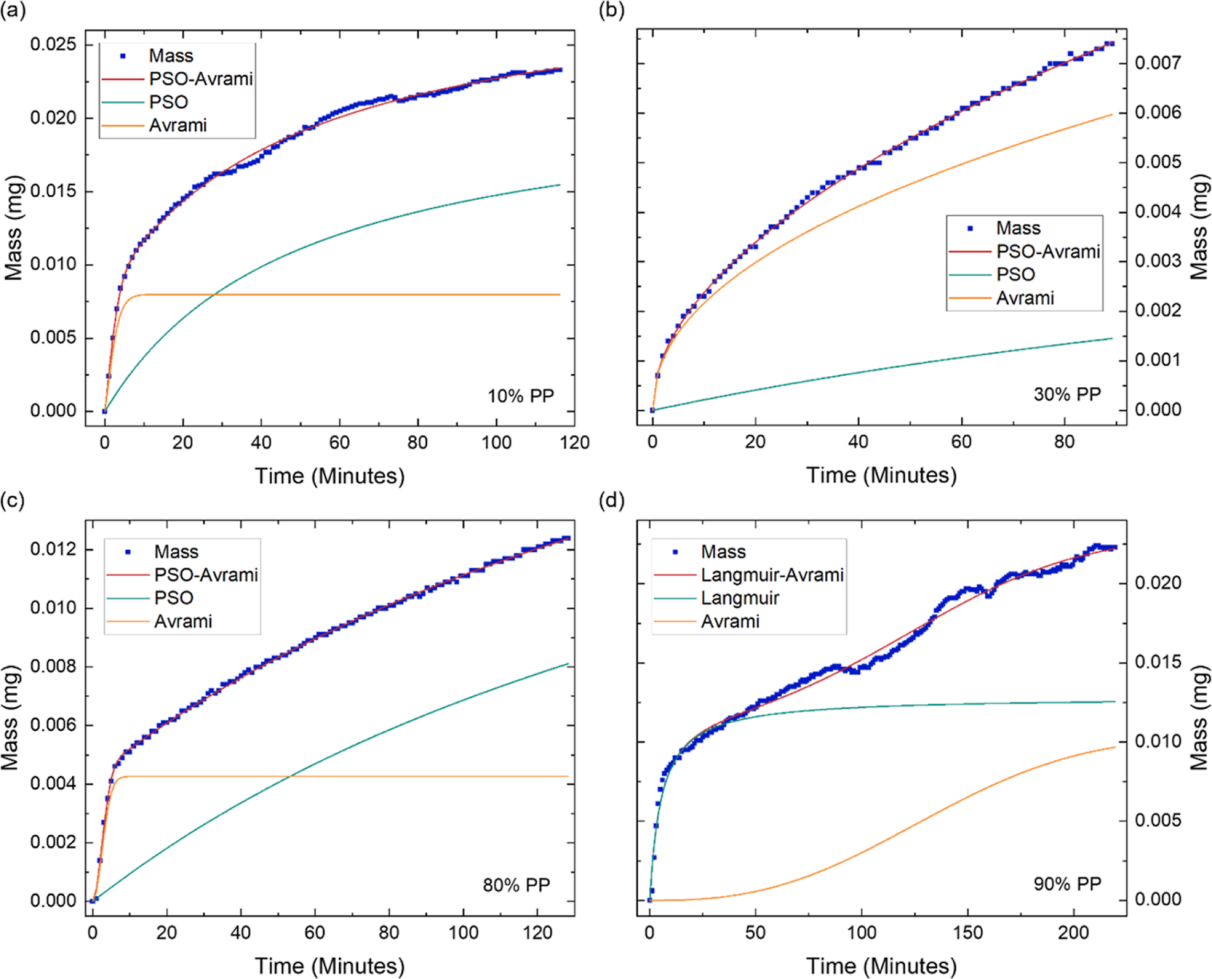
Ethanol sorption kinetics of GPA, showing the increase in mass over time at different partial pressures of (a) 10% PP, (b) 30% PP, (c) 80% PP and (d) 90% PP. The starting mass and time have been shifted to zero and the fit of the hybrid models and their individual components is displayed. The presented data enables the in-depth investigation and discussion of GPA’s ethanol sorption kinetics.

Analyzing the sorption kinetics at 80% PP and below, ethanol sorption is best modeled with a PSO-Avrami hybrid model. The gradual sorption shown by the PSO model demonstrates the gradual chemisorption of the adsorbent to the adsorbate, specifically hydrogen bonding between the hydroxyl groups on GO with the hydroxyl group on ethanol.^28^ Unlike with water sorption, the Avrami exponent does not remain constant throughout the sorption process. Between 10 and 20% PP (Figure 6a), *n* = 1.2 ± 0.1, suggesting a 1D growth mechanism. This is facilitated by the nucleation of GO’s functional groups through its interaction with ethanol, shown by the PSO component dominating the sorption mechanism. However, at 30% PP (Figure 6b), there is a sudden decrease in the contribution of the PSO component, with the Avrami component dominating sorption, and the corresponding exponent decrease to 0.5, indicating no nucleation and instead, the sorption is driven by the diffusion of ethanol into the porous structure.^53^ Between 40 and 80% PP (Figure 6c), the PSO component yields the slower, but more dominant sorption mechanism and the Avrami exponent gradually increases from *n* = 0.74 to *n* = 0.95. The gradual diffusion of ethanol into GPA enables the vapor to interact with more functional groups, increasing GO-ethanol interactions in an inhomogeneous, time-dependent manner.^53,54^

At 90% PP, the optimal model shifts to the Langmuir–Avrami model (Figure 6d). The Langmuir model shows initial rapid sorption which plateaus rapidly, suggesting the occupation of GPA’s functional groups. As the Langmuir component plateaus, the contribution of the Avrami component starts to increase. The Avrami exponent of *n* = 2.2 indicates a more complex, 2D growth mechanism.^55^ As the functional groups have now been occupied, the continued sorption of ethanol relies on ethanol–ethanol bonding. High concentrations of ethanol in the vapor phase are known to initiate increased association between ethanol molecules and create clusters of ethanol molecules.^56^ These complex sorption kinetics, and the absence of any observed liquid condensation, indicates that the GPA continues to function as an absorber at high partial pressures, rather than a condensation surface.

**Figure 7:**
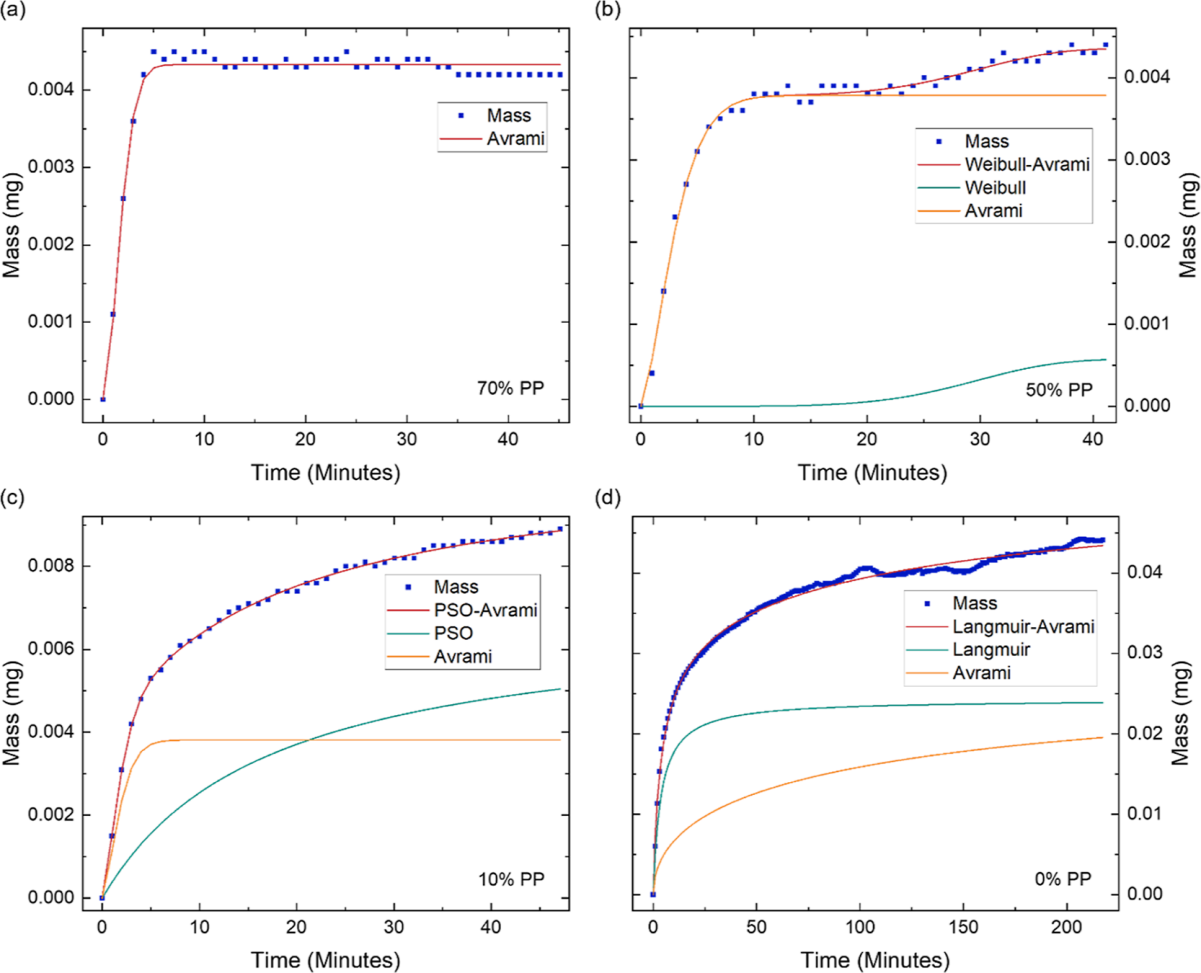
Ethanol desorption kinetics of GPA, illustrating the mass decrease over time at different partial pressures of (a) 70% PP, (b) 50% PP, (c) 10% PP and (d) 0% PP. The initial mass and time have been shifted to zero. The mass values are multiplied by −1 for easier comparison with the adsorption kinetics. The fit of the hybrid models is displayed along with the individual components. The presented data allows for the in-depth investigation of GPA’s ethanol desorption kinetics.

Analyzing the desorption characteristics, the PP between 80 and 60% are best represented by the Avrami model (Figure 7a), which can be attributed to the release of ethanol molecules from the ethanol clusters and denucleation.^57^ Between 50 and 20% PP, the optimal desorption model is the Weibull–Avrami, with the Avrami model remaining dominant (Figure 7b). The Weibull component does not contribute toward desorption until the latter half of each desorption stage, where its influence gradually increases before plateauing, though it remains less significant than the Avrami component.

The delayed activation of the Weibull component reflects the heterogeneity of desorption. The weaker bound ethanol molecules at easily accessible sites desorb first, followed by the desorption of more tightly bound ethanol molecules or those from deeper within the porous structure. At 10% PP, desorption is best modeled by the PSO-Avrami model (Figure 7c). The Avrami model continues to drive rapid desorption, however, the contribution of the slower PSO component gradually increases and overtakes the Avrami component by the end of the PP stage, showing the influence of the ethanol molecules desorbing from GO’s hydroxyl groups. At 0% PP, the optimal model changes to the Langmuir–Avrami model (Figure 7d), suggesting that the rate of desorption is proportional to the concentration of ethanol adsorbed.

### 2.3 Acetone Sorption

**Figure 8:**
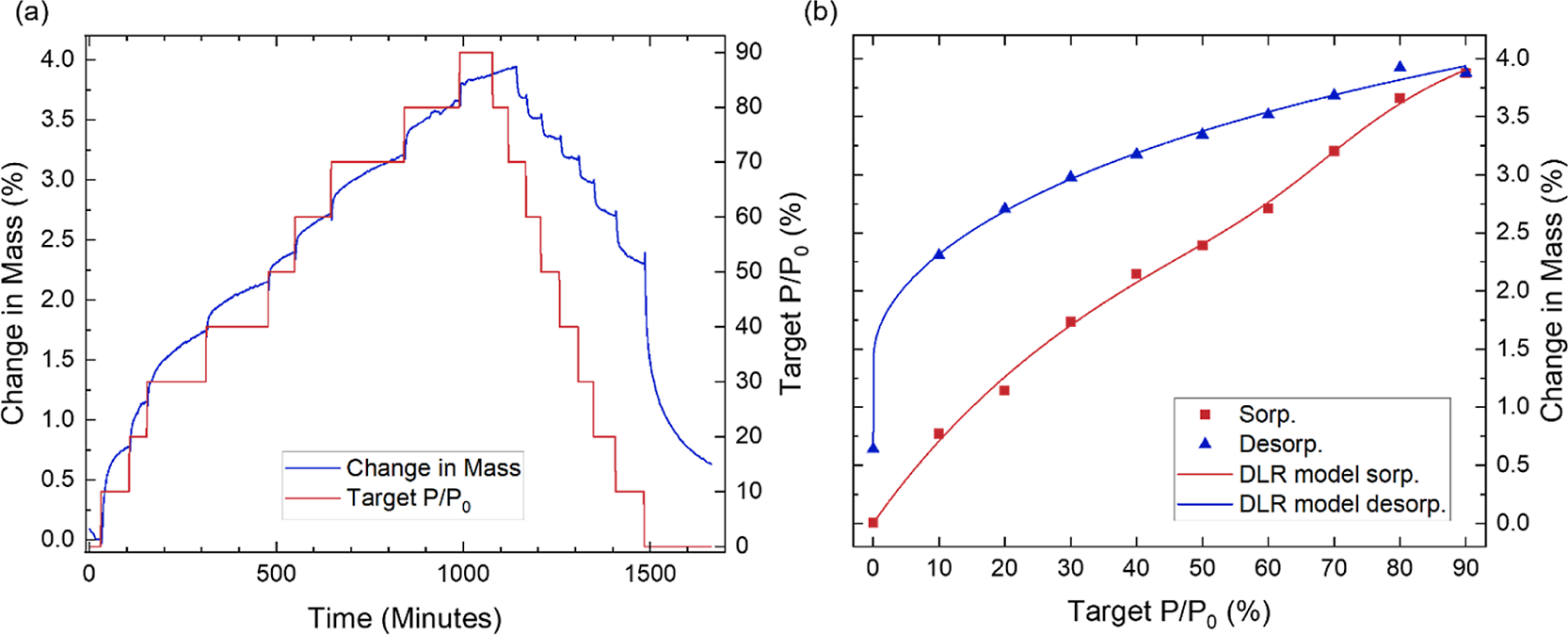
(a) Percentage change in mass of GPA over time as the partial pressure of acetone vapor is varied between 0 and 90%. (b) Resulting isotherm illustrating the adsorption behavior. The presented data facilitate the investigation of the kinetics, isotherms and hysteresis behaviors of acetone sorption on GPA.

Acetone sorption exhibits a similar maximum capacity to ethanol, reaching 4.3 ± 0.1% (0.043 ± 0.001 g/g) at 90% PP (Figure 8a). The bonding of the acetone to GPA, attributed to dipole–dipole interactions between the hydroxyl groups on GO with the carbonyl groups on acetone, leaves alkyl groups on the surface of GPA, such that further sorption of acetone is reliant on weak van der Waals forces between the adsorbed acetone molecules and the additional acetone molecules.^58^ This maximum sorption capacity is lower than that of other porous carbon-based materials.^59,60^ Previous studies have shown that the optimal pore size for acetone sorption falls within the micropore range, specifically between 2 and 5 times the diameter of an acetone molecule.^58^ Despite this, there is potential to modify GPA to enhance its properties for acetone sorption applications through nitrogen doping and reducing the pore size to the micropore range.^61,62^ Counterintuitively, Figure 8a also shows that the mass of GPA increases when the PP reduces to 80%. This can be attributed to the mass not reaching equilibrium at 90% PP. With such small mass changes at each PP, a more stringent stop criterion than less than 0.002% mass change per minute for 10 min is required.

Similar to ethanol sorption, the resulting isotherm demonstrates type IVa behavior, with hysteresis, due to the capillary condensation and the DLR model, showing the best fit (Figure 8b). The lower mass variation of 0.4% between the start of the sorption cycle and the end of the desorption cycle originates from the weaker GO-acetone bonds compared to GO-ethanol bonds, allowing the more reversible acetone sorption.

**Figure 9:**
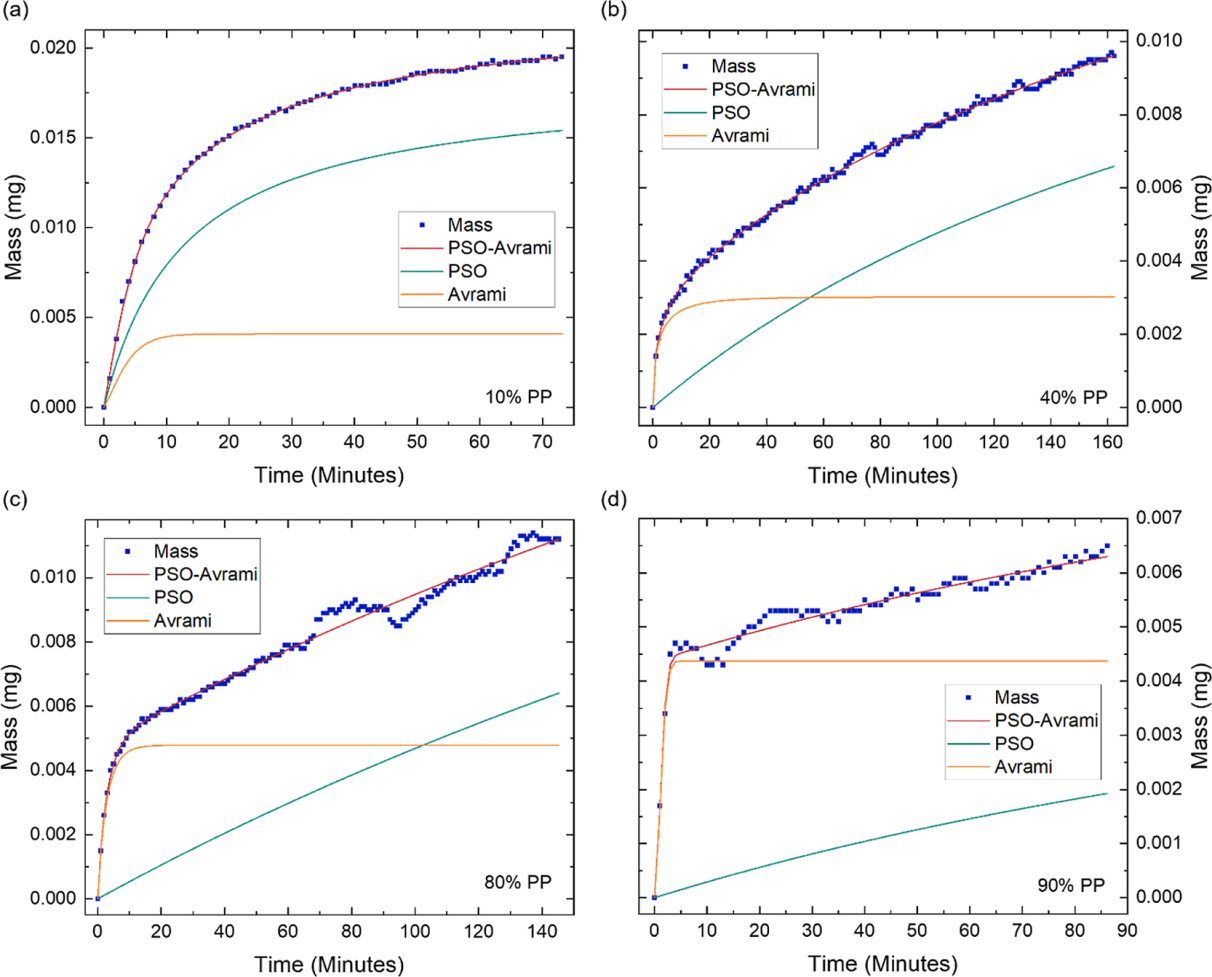
Acetone sorption kinetics of GPA, showing the increase in mass over time at different partial pressures of (a) 10% PP, (b) 50% PP, (c) 80% PP and (d) 90% PP. The starting mass and time have been shifted to zero, and the fit of the hybrid models and their individual components are displayed.

The sorption of acetone up to 80% PP is similar to ethanol sorption and is best represented by the PSO-Avrami model. The PSO model remains dominant throughout, showing the significant influence of the interactions between GPA and acetone, facilitated by dipole–dipole interactions between acetone and GO’s functional groups.^63^ Between 10 and 30% PP (Figure 9a), *n* = 1.2 ± 0.1, suggesting a 1D growth mechanism from the nucleation of GO’s functional groups. This reduces to 0.54 at 40% PP (Figure 9b), indicating the diffusion of acetone into GPA without any nucleation. The Avrami exponent then increases to 0.96 ± 0.07 between 50 and 80% PP (Figure 9c), implying the heterogeneous, time-dependent nucleation of GO’s functional groups.

The sorption behavior of ethanol and acetone differ at 90% PP, where the optimal fit for acetone remains the PSO-Avrami model (Figure 9d), suggesting that monolayer sorption has not been achieved, due to the weak GO-acetone interactions.^28^ Although the Avrami component does increase at 90% PP, it only reaches *n* = 1.8. This reflects the ongoing nucleation of GO’s functional groups as the acetone molecules continue to diffuse through the structure.^53^ Acetone clusters are much less likely to form compared to ethanol clusters, as they rely on weaker van der Waals forces and methyl-carbonyl group interactions.^64^

**Figure 10:**
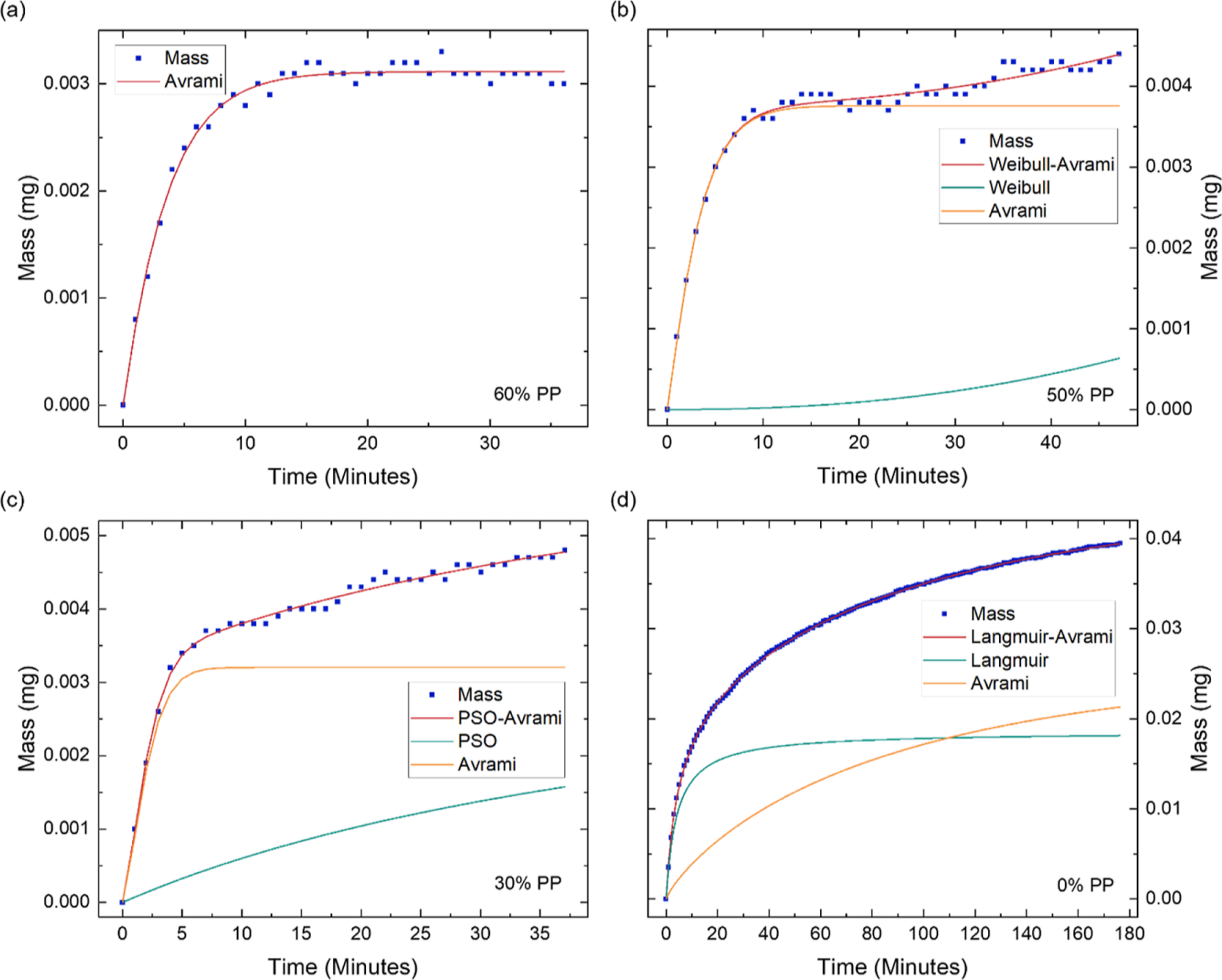
Acetone desorption kinetics of GPA, illustrating the mass decrease over time at different partial pressures of (a) 60% PP, (b) 50% PP, (c) 30% PP and (d) 0% PP. The initial mass and time have been shifted to zero. The mass values are multiplied by −1 for easier comparison with the adsorption kinetics. The fit of the hybrid models is displayed along with their individual components. The presented data allows for the in-depth investigation of GPA’s acetone desorption kinetics.

Negating the increase in mass observed as the PP is decreased to 80%, due to an equilibrium not being reached, acetone desorption shows similar characteristics to ethanol desorption. At 70% PP, the optimal fit is the Avrami model (Figure 10a), originating from denucleation. This transitions to the Weibull–Avrami model between 60 and 40% PP (Figure 10b), with the Avrami component being the dominant sorption mechanism and the low, delayed contribution of the Weibull model representing the heterogeneity of desorption from different binding sites and different locations in the porous structure.

As the PP drops to 30%, the optimal model transitions to the PSO-Avrami model (Figure 10c). The PSO model shows the gradual dechemisorption of the acetone molecules from GPA, which becomes more significant as the PP is further reduced to 10%. Finally, once the PP decreases to 0% (Figure 10d), the optimal model becomes the Langmuir–Avrami model, implying that the rate of desorption is proportional to the concentration of acetone molecules adsorbed.

Our findings highlight that the sorption capacities of GPA for ethanol and acetone vapors observed in this study represent only a fraction of its potential total capacity. The slow, continuous adsorption process, which remained unsaturated within the experimental time frame, indicates that GPA may possess a significantly higher capacity than initially apparent. Fully quantifying this capacity would require longer experimental durations tailored to specific application timeframes, which was beyond the scope of this work. Therefore, while GPA exhibits promising sorption behavior, a direct comparison with existing adsorbent materials was not undertaken. Future research would aim to determine the equilibrium capacities over extended periods and evaluate GPA’s performance against other adsorbents under standardized conditions to comprehensively assess its effectiveness for specific applications.

## 3 Conclusions

The sorption characteristics of GraPhage13 aerogels with water, ethanol and acetone have been comprehensively characterized through in-depth DVS studies with the detailed analysis of isotherms and sorption–desorption kinetics. GPA demonstrates a remarkably high sorption capacity of 0.68 g/g, twice that of traditional silica gel adsorbents and 20% higher than GO laminates. This superior performance is attributed to its high surface area, extensive porous network and the strong interactions between water molecules and the functional groups on the GO within GPA, along with water–water interactions that enable indefinite multilayer adsorption. The low level of hysteresis and the stability of GPA under consecutive sorption–desorption cycles underscore the reversibility of water sorption and the reusability of GPA. GPA exhibits a lower sorption capacity for ethanol and acetone. This is largely due to the finite nature of multilayer adsorption, slower sorption kinetics resulting from the lower affinity of ethanol and acetone for GPA and the significant hysteresis as result of the capillary condensation. Nevertheless, the *tuneability* of GPA offers substantial potential for enhancing acetone sorption capacity and its ethanol sorption capacity is comparable or superior to that of similar porous carbon-based materials, highlighting GPA’s versatility in adsorbing a wide range of vapors.

This study underscores the remarkable sorption characteristics of GPA, demonstrating both its capability and versatility. Collectively, the findings pave the way for the development and integration of GPA into advanced graphene-based devices for environmental monitoring, freshwater generation and miniaturized biomedical sensors. Realizing these applications on an industrial scale will require advancing the production process from laboratory methods to efficient and cost-effective industrial techniques. Moreover, integrating GPA into graphene-based devices will be critical for unlocking its potential across a diverse range of applications. While challenges remain in transitioning from laboratory-scale processes to industrial settings, ongoing advancements in bioengineering (e.g., enhancing M13 bacteriophage yield) and material processing (e.g., continuous flow systems for GO reduction) are promising. These innovations align with the principles of green chemistry, which are central to GPA production, and support its industrial feasibility.

## 4 Materials and Methods

### 4.1 Propagation and Purification of M13 Bacteriophage

The method for culturing and purifying the M13 bacteriophage is given in.^65^ Initially, One Shot TOP10F′ Chemically Competent *Escherichia coli* (Thermo Fisher Scientific) was cultivated on nutrient agar plates overnight at 37 °C. The bacteria were then transferred to 50 mL falcon tubes containing nutrient broth (NB) and tetracycline (Sigma) to achieve a final concentration of 5 μg/mL and incubated overnight at 37 °C with shaking at 150 rpm. The M13 phage was incubated overnight in NB supplemented with the *E. coli*-NB-tetracycline solution, and tetracycline at a final concentration of 5 μg/mL. The resulting solution underwent centrifugation twice, followed by mixing with a solution of 25% polyethylene glycol (PEG) 6000 and 2.5 M NaCl and stirring on ice for 90 min. After centrifugation, a white pellet containing M13 was obtained, which was then resuspended in deionized water (DIW). This solution was further processed through centrifugation, and the resulting supernatant was subjected to another round of PEG + NaCl treatment and centrifugation. Finally, a white pellet containing purified M13 was obtained and resuspended in DIW.

### 4.2 UV–Vis Spectroscopy

The concentration of M13 in DIW was assessed using a UV–vis spectrophotometer (Aligent Cary 60 UV–vis) equipped with a 1 cm light path quartz cuvette. Initially, a spectrum of DIW was obtained as a reference. Samples were transferred to a 1.5 mL centrifuge tube and placed on an orbital shaker for 1 min to ensure homogeneity. M13 concentration was determined using the Beer–Lambert Law with an extinction coefficient of 3.84 cm^2^mg^–1^ at 269 nm. Phage viability was confirmed by specific spectral characteristics: an absorbance peak at 269 nm (A_269_), a local minimum absorbance at 245 nm (A_245_), and a baseline at 350 nm (A_350_). Purity and viability were verified by an A_269_/A_245_ ratio of ∼1.37 and an A_350_/A_269_ ratio of ∼0.02.^65^

### 4.3 Fabrication of GraPhage13 Aerogels (GPA)

The method for creating GraPhage13 aerogels (GPAs) follows Passaretti et al., involving the addition of M13 and GO to a 10 mM citrate buffer pH 4.9.^12^ This induces self-assembly, forming an aggregate. After mixing and centrifugation, a GO-M13 pellet was obtained from which 90% of the supernatant was removed and the GO-M13 resuspended create the GraPhage13 hydrogel (GPH). 350 μL of GPH was deposited onto 16 mm glass coverslips and dried in a vacuum for 120 min to a final pressure of approximately 0.05 mbar, yielding GPA.

### 4.4 Scanning Electron Microscopy (SEM)

The morphology of GPA was observed with a Hitachi SU5000. Due to the insulating nature of the GPAs, SEM images were taken at 0.5 kV to reduce charge build-up.

### 4.5 Dynamic Vapor Sorption

Sorption measurements were performed with a DVS Resolution, at a constant temperature of 25 °C and flow rate of 200 mL/min. The partial pressure (PP) of water, ethanol and acetone were varied from 0% to 90% to 0% in increments of 10%. The stop criterion for each stage was defined as a mass change of less than 0.002% per minute, stable over a period of 10 min,^20^ for a minimum duration of 30 min and a maximum of 12 h. This process was repeated with at least three GPA samples using the DVS technique to ensure reproducibility of the sorption measurements.

### 4.6 Model Selection and Bayesian Information Criterion (BIC)

The optimal model for the isotherms and the sorption kinetics at each PP stage were determined using Python, by testing various models and calculating their Bayesian Information Criterion (BIC) value
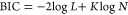
1Here, *L* is the likelihood, *K* is the number of parameters and *N* is the number of data points.^66^ The BIC provides a balance between the goodness of fit and model complexity by penalising the addition of parameters, which is not included in the traditional least-squares fitting.

The isotherms were fitted with each isotherm model, the BIC was calculated and the model with the lowest BIC value was considered as the best fit. Similarly, each kinetic model was fitted to the experimental data for each sorption and desorption stage and the model with the lowest BIC value was selected, When the difference in BIC values was less than 6, indicating statistically similarity, factors such as the physical significance of the fitted parameters, consistency with the adsorption mechanism expected at the PP stage, and the alignment with parameters from preceding or subsequent stages, were taken into account.

A brief introduction to each kinetic model, including their equations and physical processes, are provided below.

- Langmuir model: this describes monolayer adsorption onto a surface with a finite number of identical sites, with kinetics proportional to the concentration of adsorbed molecules. It assumes that once a site is occupied, no further adsorption can occur at that site. It is given by

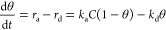
2where  is the net adsorption rate, θ is ratio of the adsorbed amount per mass of adsorbent compared to its maximum adsorption capacity, *t* is time, *r*_a_ and *r*_d_ are the rate of adsorption and desorption respectively, *k*_a_ and *k*_d_ are the adsorption and desorption rate constants respectively, and *C* is the concentration of adsorbate.^67^

- Pseudo-Second Order (PSO) model: the PSO model is used to describe chemisorption kinetics where the rate of occupation of adsorption sites is proportional to the square of the number of unoccupied sites. It is given by

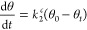
3where *k*_2_^*c*^ is the PSO rate constant, and θ_0_ and θ are the amount adsorbed at equilibrium and time *t*, respectively.^68^

- Avrami model: this describes adsorption kinetics involving nucleation and growth processes. It is given by

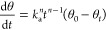
4where *k*_a_ is the Avrami kinetic constant and *n* is the Avrami constant, reflecting the dimensionality of growth.^41^

- Weibull model: this is a statistical distribution used to describe the time until a particular event occurs. It is applicable to adsorption kinetics with a distribution of adsorption energies, accounting for heterogeneity in adsorption sites. It is given by

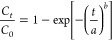
5where *C*_*t*_ is the adsorbate concentration at time *t*, *C*_0_ is the feed adsorbate concentration of adsorbate, *a* is the rate parameter and *b* is the shape parameter.^69^

- Intra-particle diffusion model: this describes adsorption as a process controlled by the diffusion of adsorbate molecules into the pores of the adsorbent. It is given by

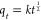
6where *q*_*t*_ is the adsorption capacity at time *t* and *k* is the kinetics parameter.^70^
